# Robot-assisted closed reduction and navigated fixation for pelvic ring injuries: a focused narrative review of a reduction-fixation-audit framework

**DOI:** 10.3389/fmed.2026.1883771

**Published:** 2026-08-27

**Authors:** En Wu, Feng Gu, Wenkai Zhang, Xiaoyu Li, Ye Liang, Zhanxiao Liu, Shen Liu, Yonghui Liang

**Affiliations:** Department of Emergency, Aerospace Center Hospital, Beijing, China

**Keywords:** minimally invasive surgery, pelvic ring fracture, percutaneous fixation, postoperative audit, precision medicine, robot-assisted fracture reduction, Rossum robot, surgical navigation

## Abstract

Pelvic ring injuries combine three-dimensional deformity with narrow osseous corridors and proximity to neurovascular structures. Minimally invasive fixation can reduce exposure-related morbidity, but its success remains constrained by the quality of reduction achieved before fixation. This focused narrative review examined CT-based phenotyping and planning, mirror-template reconstruction, robot-assisted fracture reduction (RAFR), navigated percutaneous fixation, and postoperative audit. English- and Chinese-language literature was identified by targeted searches from database inception to May 31, 2026. Evidence was synthesized by study design and maturity; no pooled effect estimate or formal risk-of-bias score was undertaken. The published RAFR literature consists predominantly of model validation, a first-in-human report, small single-center series, and one non-randomized comparison. These studies demonstrate technical feasibility and early radiographic acceptability in selected fresh unstable pelvic ring injuries. A 2024 series extends direct but still preliminary evidence to displaced fragility fractures. Recruitment centers, investigators, and enrollment periods overlap across several reports, so cohort independence cannot be assumed. Reported reductions in blood loss, incision length, radiation exposure, or early functional scores should therefore be treated as associations rather than proof of comparative effectiveness. Long-term safety, durable function, learning curve, multicenter reproducibility, and cost-effectiveness remain unestablished. The main contribution of this review is a surgeon-in-the-loop reduction-fixation-audit framework that separates four endpoints: technical feasibility, radiographic reduction, clinical effectiveness, and safety. It also proposes candidate reporting domains and distinguishes evidence-informed early-use scenarios from investigational applications. Force-controlled stress testing for equivocal instability remains a research hypothesis, not an established indication.

## Highlights

- Minimally invasive pelvic fixation is constrained by reduction quality as well as screw accuracy.
- Navigated or robot-assisted screw placement addresses implant trajectory; it does not substitute for pelvic reduction.
- Published RAFR studies support selected technical feasibility and early radiographic acceptability, not proven clinical superiority.
- The current clinical evidence is largely Rossum-device-specific and should not be generalized to all reduction robots.
- Reports with overlapping investigators, centers, and recruitment periods should not be counted as independent cohorts without patient-level confirmation.
- Future studies should separately report technical feasibility, CT reduction, implant accuracy, clinical effectiveness, safety, learning curve, and cost-effectiveness.
- Force-controlled stress testing for equivocal pelvic instability remains a research hypothesis and is not an established indication.

## 1 Introduction

Pelvic ring fractures range from low-energy osteoporotic fragility fractures to high-energy disruptions associated with hemorrhage, visceral injury, neurological deficit, and long-term disability. Surgical treatment must restore pelvic symmetry and posterior ring stability while limiting physiological burden. This balance makes pelvic ring surgery a setting in which decisions are shaped by fracture morphology, patient physiology, bone quality, and individual anatomy, rather than by classification alone.

A patient-specific, image-guided approach rests on three interacting phenotypes. The fracture phenotype describes the injured ring in three dimensions, including displacement direction, posterior and anterior ring involvement, sacral morphology, reducibility, and the dominant reduction vector. Tile/AO and Young-Burgess classifications remain useful shorthand, but injuries within the same category may require different maneuvers (1). The patient phenotype includes physiology, frailty, osteoporosis, soft-tissue status, neurological injury, and readiness for definitive surgery. The anatomical phenotype determines whether percutaneous fixation is feasible because sacral dysmorphism, comminution, bone quality, and residual displacement can narrow or distort safe corridors.

This patient-specific logic leads to a practical problem familiar to pelvic surgeons: in minimally invasive surgery, reduction is often the bottleneck. Closed reduction may involve longitudinal traction, Schanz pin manipulation, external fixation, pelvic frames, clamps, staged maneuvers, or limited-open assistance. These maneuvers are frequently guided by two-dimensional fluoroscopy despite a three-dimensional deformity. Residual malreduction can narrow osseous corridors, increase cortical breach risk, weaken stability, and contribute to malunion, pain, or gait disturbance. Conversely, extensive open reduction may increase blood loss, wound morbidity, and physiological stress, particularly in polytrauma or frail elderly patients.

Minimally invasive pelvic fracture surgery has progressed from fluoroscopy-guided percutaneous fixation to computer-assisted navigation and robotic screw placement. Percutaneous iliosacral screws, trans-sacral trans-iliac screws, anterior ring screws, anterior column screws, and internal fixation devices can reduce soft-tissue dissection and blood loss compared with extensile open approaches. However, minimally invasive fixation does not remove the need for reduction. Safe screw placement often depends on restoring the displaced hemipelvis, sacroiliac joint, sacral fracture, or anterior ring sufficiently to recreate the osseous corridor (2–4).

Most navigation and screw-placement robotic systems were designed around fixation. They can improve trajectory accuracy and reduce repeated fluoroscopic adjustment, but they do not by themselves solve three-dimensional reduction. Recent scoping and systematic reviews likewise describe a literature dominated by small cohorts, heterogeneous technologies, and limited comparative follow-up (2, 5, 6).

Dedicated RAFR systems have emerged to address this upstream step. Rather than beginning with the screw trajectory, these systems move robotic assistance to the reduction phase. Representative platforms combine preoperative CT-based three-dimensional planning, mirror-template reconstruction, intraoperative cone-beam CT (CBCT) registration, optical tracking, passive holding devices, elastic traction, and force-position-monitored robotic manipulation (3, 7–13).

Existing reviews provide complementary foundations. Kou et al. summarized the engineering evolution of robot-assisted fracture-reduction systems (3). Shen et al. described the broader evolution from open to conventional closed, navigation-assisted, and robot-assisted pelvic reduction (14). Wu et al. focused on robot-assisted pelvic fracture fixation (2), whereas Le Baron et al. systematically reviewed operating-room technologies across pelvic and acetabular trauma (5). A 2026 review also surveyed robotic management of complex pelvic fractures (15). The present review differs by linking reduction planning, surgeon-supervised execution, navigated fixation, and postoperative audit, while explicitly separating feasibility, radiographic accuracy, clinical effectiveness, and safety. Their scope and differences are summarized neutrally in Supplementary Table S1.

The value of this review is conceptual synthesis rather than pooled effect estimation. It develops a reduction-fixation-audit framework, identifies tentative selection boundaries, and proposes outcome domains for future validation. Because the evidence is immature and partly device-specific, the framework should not be interpreted as proof of superiority or as a clinical guideline.

## 2 Scope and literature selection

This focused narrative review addresses patient-specific, image-guided minimally invasive surgery for pelvic ring injuries. It examines how CT-based phenotyping, reduction planning, robot-assisted or otherwise controlled closed reduction, navigated percutaneous fixation, and postoperative assessment can be organized into a reduction-fixation-audit pathway. Acetabular and proximal femoral fractures were excluded as main topics because their reduction goals, implants, corridors, and outcome measures differ substantially.

Targeted searches were performed in PubMed, Web of Science, Frontiers, SpringerLink, ScienceDirect, CNKI, WanFang, and SinoMed from database inception to May 31, 2026. English- and Chinese-language publications were considered. Database-appropriate combinations were iteratively built from terms for pelvic ring injury, fracture reduction, robot-assisted or autonomous reduction, Rossum Robot, TiRobot, navigation, O-arm/three-dimensional imaging, percutaneous screws, mirror reconstruction, occult instability, Matta grading, and Majeed outcomes. Reference lists of relevant reviews and clinical reports were searched manually.

Eligible publications addressed at least one element of the framework: CT-based planning, mirror-template or computational reconstruction, robot-assisted reduction, navigation- or robot-assisted percutaneous pelvic fixation, standardized assessment of reduction or implant position, pelvic ring outcomes, or force-controlled stability assessment. Foundational classification and outcome papers were retained. Studies centered on acetabular or proximal femoral fractures, non-pelvic reduction, industrial robotics without a pelvic trauma application, or implant design without a reduction/navigation component were excluded from the main synthesis.

Two authors independently screened titles, abstracts, and accessible full texts, with disagreements resolved with the senior author. Because the search was iterative and narrative, exact query strings and a PRISMA-style record flow were not prospectively recorded. Formal risk-of-bias scoring and pooled effect estimation were not performed. Evidence was interpreted by design and maturity, with caution for preclinical, single-case, single-center, retrospective, and device-specific reports. These choices limit reproducibility and make the review framework-generating rather than systematic.

Data extraction focused on study design, setting, sample size, recruitment period, device and workflow, reduction metrics, functional outcomes, follow-up, adverse events, and implementation measures. Author lists, institutions, and enrollment periods were cross-checked for possible overlapping cohorts. When patient-level independence could not be verified, cohorts were not summed and possible overlap was explicitly flagged.

## 3 Digital planning for patient-specific reduction

**Figure 1:**
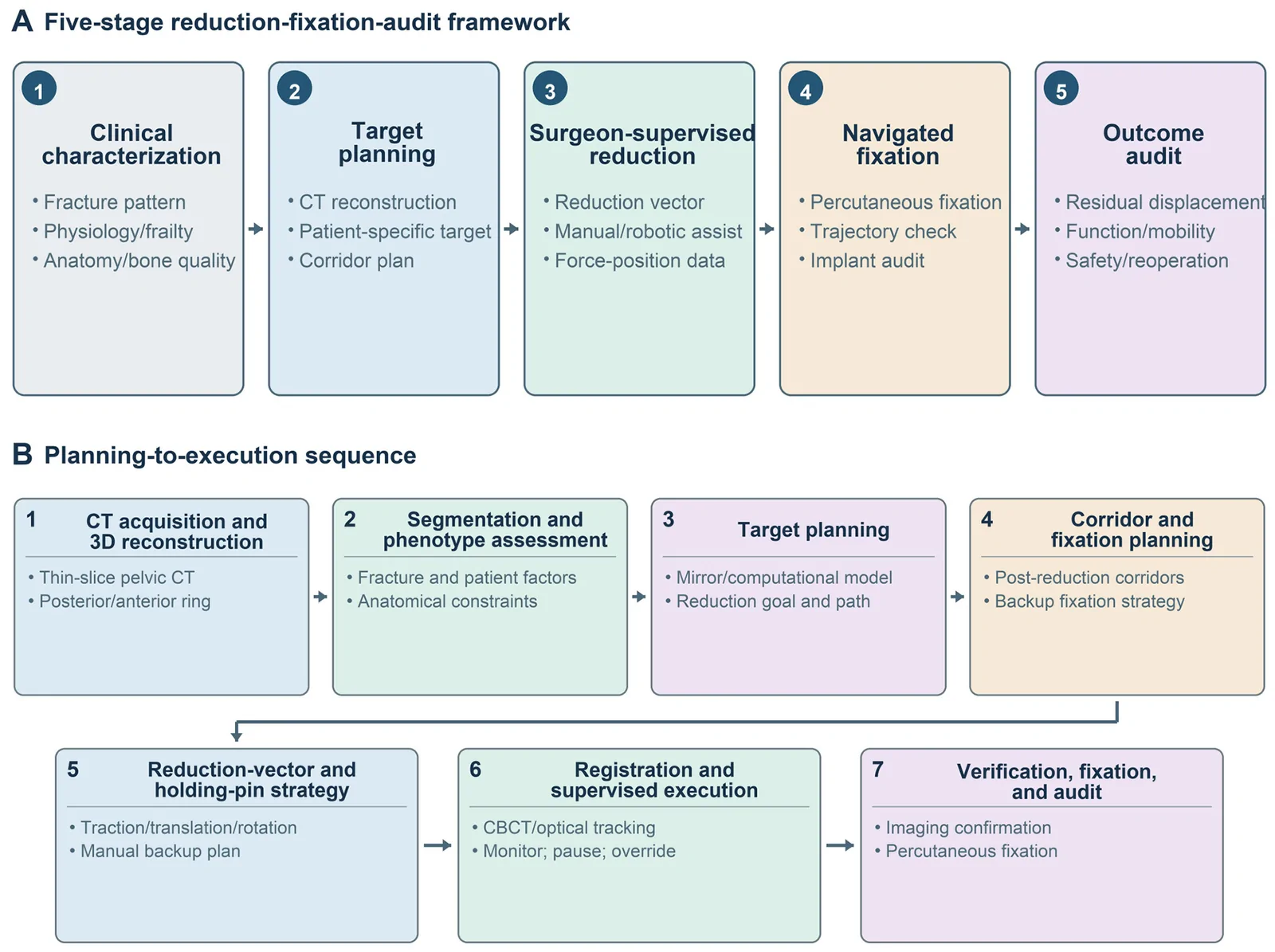
Patient-specific reduction-fixation-audit framework and surgeon-supervised planning-to-execution sequence for pelvic ring surgery. **(A)** links clinical characterization, target planning, surgeon-supervised reduction, navigated fixation, and outcome audit. **(B)** expands the planning-to-execution sequence from CT acquisition and phenotype assessment to target/corridor planning, reduction-vector and holding-pin strategy, registration, monitored execution with pause or override, imaging verification, fixation, and postoperative audit. The framework is conceptual and is not a device-specific protocol or clinical guideline.

For readers more familiar with open or fluoroscopy-guided reduction, Figure 1 provides a visual summary of the digital planning and execution sequence and shows how it links to intraoperative robot-assisted reduction.

### 3.1 CT-based three-dimensional reconstruction

Digital planning starts with high-quality CT and three-dimensional reconstruction. Compared with plain radiographs or two-dimensional fluoroscopy, CT directly shows posterior ring displacement, sacral fracture morphology, sacroiliac incongruity, anterior ring disruption, comminution, and potential screw corridors. In a patient-specific, image-guided pathway, CT informs diagnosis, reduction planning, virtual implant trajectories, intraoperative navigation, and postoperative audit.

In conventional practice, surgeons infer three-dimensional deformity from inlet, outlet, anteroposterior, and lateral fluoroscopic views. Digital reconstruction makes that reasoning explicit: fragments can be segmented, displacement vectors visualized, and target alignment estimated before the patient enters the operating room.

### 3.2 Mirror-template reconstruction

Mirror-template reconstruction is a useful tool for patient-specific reduction planning in unilateral pelvic ring injuries. Because pre-injury CT is rarely available, the intact or less-injured hemipelvis can be mirrored to estimate the target position of the injured side. Ead et al. (16) described virtual reconstruction of unilateral pelvic fractures using pelvic symmetry, and Zhao et al. (17) proposed automatic reduction planning based on pelvic symmetry.

For robot-assisted reduction, mirror-template planning is more than a visualization step. It defines a target position and reduction trajectory that can be translated into robotic execution. Its reliability decreases when the contralateral hemipelvis is injured, posterior comminution is bilateral, or pre-existing deformity or previous pelvic surgery invalidates the mirror assumption.

### 3.3 Automated segmentation and learning-based planning

Automated segmentation and learning-based planning build on manual or semi-automatic mirroring. A 2025 learning-based pipeline aimed to reduce subjective interpretation and manual planning time (18). Patient-specific musculoskeletal modeling has also been proposed to estimate soft-tissue forces and improve trajectory planning, but remains a planning study rather than clinical effectiveness evidence (19).

These methods may make planning more standardized and quantitative, but automated planning is not autonomous decision-making. Algorithms can identify fragments and estimate target anatomy; the surgeon must still judge reducibility, soft-tissue constraints, neurovascular risk, fixation strategy, and patient tolerance.

### 3.4 Reduction-fixation coupling

Reduction planning and virtual screw planning should be considered together. A trajectory that appears safe on the unreduced CT may become unnecessary or unsafe after reduction; conversely, a corridor that appears compromised before reduction may become available once alignment is restored. Planning should therefore evaluate the intended post-reduction pelvis, not only the initial displaced anatomy.

This coupling is central to robot-assisted pelvic surgery. The purpose of robotic reduction is not simply to improve a radiograph, but to restore a geometry that allows safe percutaneous fixation. Reduction and fixation are best treated as interdependent steps rather than separate technical achievements.

## 4 Robot-assisted closed reduction: the currently reported Rossum-based workflow

### 4.1 Pathway rationale and component justification

If the main contribution of this review is a reduction-fixation-audit pathway, RAFR should be judged by how well it strengthens each step of that pathway, not by the novelty of a single device. The literature supports four linked components. First, CT-based planning and mirror-template reconstruction define a patient-specific target for the displaced hemipelvis or posterior ring. Second, registration, tracking, traction, and force-position-monitored manipulation translate that target into a controlled reduction maneuver. Third, fluoroscopy or intraoperative three-dimensional imaging verifies reduction before fixation. Fourth, navigated or robot-assisted percutaneous fixation stabilizes the restored pelvic geometry, while postoperative CT-based audit separates reduction quality from implant accuracy.

The currently reported Rossum-based workflow is used as the principal clinical example because most published RAFR cohorts evaluated this platform or closely related prototypes. Device-specific results should not be generalized to an entire technology class. Accurate fixation cannot compensate for malreduction, and acceptable reduction still requires safe fixation and standardized audit.

### 4.2 System architecture mapped to the pathway

The reported workflow combines CT-based planning, fragment segmentation, mirror-template target generation, intraoperative CBCT registration, optical tracking, passive stabilization of the reference hemipelvis, elastic traction, and force-position-monitored robotic manipulation. Planning sets the target; registration links it to the patient; stabilization and traction create a controllable mechanical environment; and the robotic arm executes a constrained reduction vector while the surgeon monitors force, position, imaging, and clinical safety.

In a model-based evaluation, Zhao et al. reported image registration and fracture-reduction accuracy using an intelligent reduction robot system in pelvic fracture models (7). This supports the technical feasibility of the planning-registration-execution concept, but it remains preclinical validation rather than proof of patient-level benefit. Clinical studies should therefore be interpreted as early evidence of pathway feasibility, not as definitive evidence that RAFR is superior to conventional reduction.

### 4.3 Practical workflow of robot-assisted pelvic ring reduction

The workflow is surgeon-supervised throughout. Preoperative CT defines displacement, target alignment, and fixation corridors. Holding pins or Schanz pins connect the mobile fragment and reference hemipelvis to the reduction apparatus; intraoperative CBCT or three-dimensional imaging registers the plan to the patient. The system then applies planned translation, rotation, traction, or combined movement while force and position are monitored. The surgeon validates the target, initiates movement, judges soft-tissue and neurovascular constraints, and can pause, modify, manually assist, or abort the maneuver before imaging verification and percutaneous fixation. The planning-to-execution sequence is summarized in Figure 1.

In this review, automation means machine execution of predefined computational or mechanical tasks; autonomy means that selected planning or motion steps are executed within predefined constraints. Neither term implies unsupervised clinical decision-making. ‘Surgeon-in-the-loop' denotes continuous human validation and the ability to stop or override the system. Outcomes should separately capture technical completion, residual CT displacement, reduction trajectory error, force and torque, attempts, manual assistance, conversion, implant accuracy, adverse events, and patient-centered recovery.

### 4.4 Thematic synthesis of the available clinical evidence

**Figure 2:**
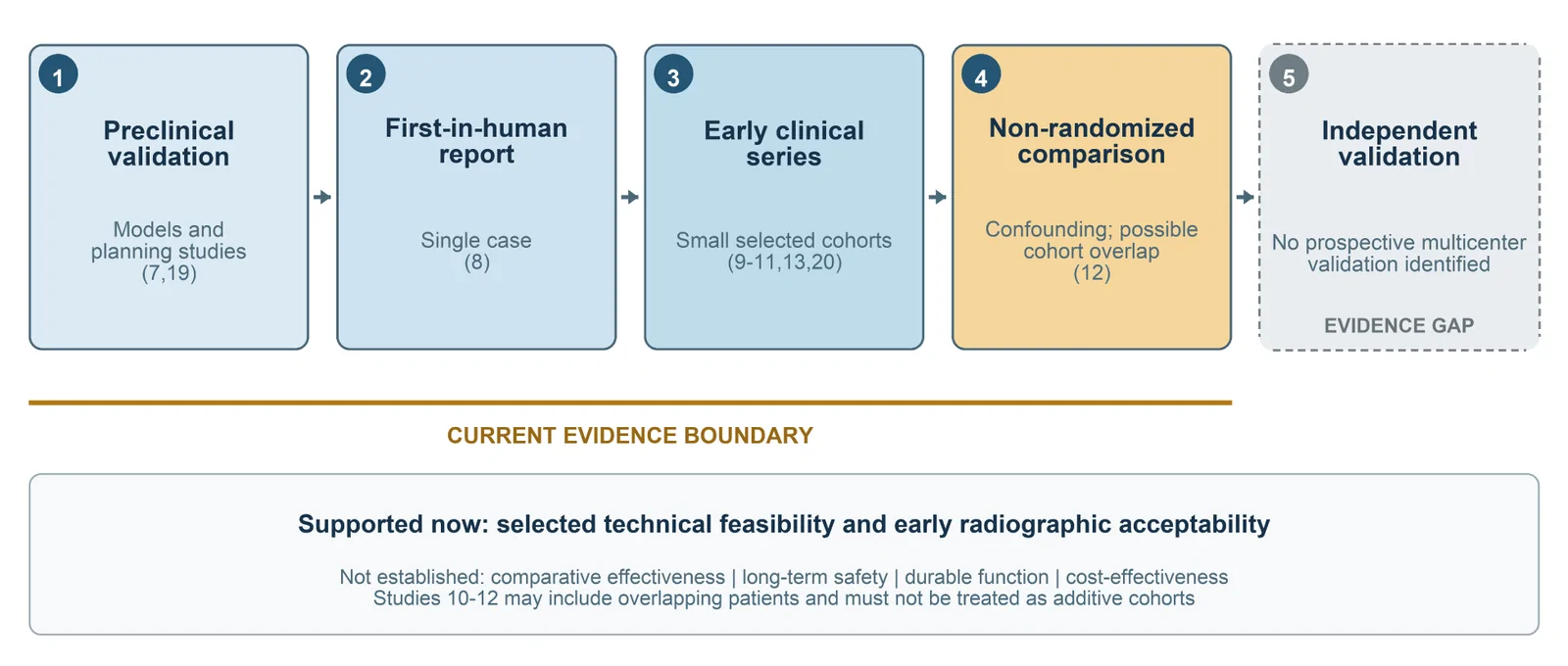
Evidence maturity and validation gaps in robot-assisted pelvic fracture reduction. Evidence has progressed from preclinical validation (7, 19) to a first-in-human case (8), small selected series (9–11, 13, 20), and one non-randomized comparison (12). Studies 10–12 may contain overlapping patients and must not be treated as additive cohorts. Independent prospective multicenter validation, rare-event safety surveillance, durable patient-centered outcomes, and cost-effectiveness remain unavailable.

Table 1 summarizes the study-level evidence. Figure 2 illustrates the maturity of this evidence and the remaining validation gaps. Model work, a first-in-human case, small clinical series, a non-randomized comparison, and a geriatric FFP case series support technical feasibility and early radiographic acceptability in selected patients (7–13, 20). These reports do not establish comparative effectiveness or long-term safety. Associations with smaller incisions, less blood loss, or early functional outcomes are vulnerable to selection, learning-curve, co-intervention, and reporting bias.

**Table 1:** Clinical and translational evidence for the currently reported robot-assisted pelvic fracture reduction workflow.

References	Evidence stage/population	Follow–up	Reported outcomes	Interpretation boundary
Zhao et al. (7)	Preclinical validation; pelvic bone models	Not applicable	Feasible image registration, path planning, and robotic reduction in controlled models.	Preclinical only; no patient–level effectiveness or safety inference.
Ge et al. (8)	First–in–human case; one Tile B2 injury	3 months	Maximum CT residual displacement 2.8 mm; Majeed 95/100; no secondary displacement, nonunion, or hardware failure reported.	Single case; no comparative or rare–event safety inference.
Zhao et al. (9)	Single–center series; 22 unstable injuries	No longitudinal clinical follow–up reported	Mean 3D point–cloud error 3.41 mm (SD 1.83); mean residual displacement 4.61 mm (SD 3.29); Matta E/G/F 16/5/1.	Immediate technical and radiographic outcomes only; no control.
Wu et al. (10)^†^	Single–center series; 20 Tile B/C injuries	No longitudinal clinical follow–up reported	Mean residual displacement 6.65 mm (SD 3.59); Matta E/G/F 7/10/3; no immediate postoperative complications reported.	Small uncontrolled sample; possible cohort overlap with studies 11 and 12.
Dai et al. (11)^†^	Retrospective series; 19 Tile B/C injuries	18/19 followed; 17 months (range 6–21)	Mean residual displacement 6.59 mm (SD 3.68); Matta E/G/F 7/9/3; final Majeed mean 86.00 (SD 6.65).	Single–center experience; possible cohort overlap with studies 10 and 12.
Dai et al. (12)^†^	Non–randomized retrospective comparison; 25 robot/25 control	Median 27 months in both groups	Robot group: mean residual displacement 7.37 mm (SD 3.44); reduction E/G/acceptable/poor 7/12/6/0; Majeed median 96 (range 87–100) vs 76 (70–100).	Selection, co–intervention, and learning–curve confounding; possible cohort overlap.
Fan et al. (13)	Single–center retrospective series; 32 fresh Tile B/C injuries	30/32 followed; mean 12 weeks	Median CT residual displacement 4.0 mm (IQR 3.0–8.0); Matta E/G/F 17/12/3; Majeed mean 76.7 (SD 12.0).	No control; short follow–up; feasibility rather than superiority evidence.
Zhao et al. (20)	Prospective single–center series; 15 displaced FFP type III injuries	6 months	Matta excellent or good in 15/15; modified Majeed mean 81.4; all fractures healed.	Selected uncontrolled geriatric cohort; comparative benefit and rare–event safety unestablished.

3D, three–dimensional; CT, computed tomography; E, excellent; F, fair; FFP, fragility fracture of the pelvis; G, good; IQR, interquartile range; SD, standard deviation. ^†^Studies 10–12 share investigators, centers, and overlapping enrollment periods; patient–level independence could not be confirmed. Outcomes are reproduced as reported and do not establish comparative effectiveness.

The most plausible early-use population is physiologically stabilized patients with fresh, closed, reducible unstable injuries, sufficient bone stock for holding pins, a usable planning reference, and a percutaneous fixation strategy. The 15-patient FFP type III series provides emerging direct evidence in older adults, but it remains single-center, uncontrolled, and limited to 6 months (20). Severe bilateral comminution, delayed irreducibility, open contamination, hemodynamic instability, unreliable mirror templates, and inadequate pin purchase remain important cautions.

Evidence independence is uncertain. Reports 10–12 share investigators, regional centers, and substantially overlapping enrollment periods; patient-level overlap could not be excluded. These cohorts must not be summed as independent observations. More broadly, most studies are small, single-center, device-specific, retrospective, or early phase, with inconsistent definitions of displacement, complications, radiation, force thresholds, conversion, and learning curve. The evidence should therefore be regarded as feasibility- and framework-generating.

### 4.5 Remaining barriers to clinical adoption

Several barriers must be resolved before RAFR can be considered a mature clinical strategy. Technical barriers include registration error, tracker or pin instability, loss of accuracy during manipulation, uncertain safe force thresholds, limited performance in osteoporotic bone, and difficulty in bilateral or highly comminuted injuries. Workflow barriers include preoperative planning time, robotic setup, intraoperative imaging requirements, staff training, troubleshooting, sterilization, and the need for an immediate backup plan for manual, limited-open, or open reduction. Evidence barriers include the absence of randomized or well-controlled multicenter studies, insufficient long-term patient-centered outcomes, unclear learning curves, and unresolved cost-effectiveness.

For now, RAFR is an early-adoption or investigational strategy rather than routine care. The Rossum pelvic fracture reduction system received marketing approval from the China NMPA in December 2023 for adult pelvic fracture reduction and navigation-related functions; authorization may differ by jurisdiction, and regulatory approval does not establish comparative clinical benefit (21). Clinical use should therefore follow local authorization, governance, training, and post-market surveillance requirements.

### 4.6 Combined reduction and fixation robotic workflow

A practical development is the combination of a reduction robot for pelvic alignment with a navigation robot for percutaneous fixation. In this sequence, RAFR restores pelvic ring morphology, and navigated fixation stabilizes the restored configuration through patient-specific corridors. The field is therefore moving from single-task robotic assistance toward an integrated reduction-fixation pathway.

Dai et al. reported a reduction-robot plus TiRobot workflow (11), and a later non-randomized comparison found associations with less blood loss and shorter incisions but longer operative time than open reduction and plating (12). Selection bias, learning-curve effects, different fixation approaches, possible cohort overlap with reports 10–11, and limited generalizability preclude causal conclusions.

## 5 Navigated fixation after robotic reduction

### 5.1 Complementary relationship

Robot-assisted reduction and navigated fixation are complementary technologies. Reduction robots aim to restore alignment; navigation or screw-placement systems improve implant trajectory. In the proposed sequence, navigated fixation is the technical extension of a successful reduction, not an isolated accuracy tool.

This distinction matters when outcomes are interpreted. A precisely inserted screw in a malreduced pelvis may still be mechanically unsatisfactory. Conversely, a good reduction can fail if fixation is unsafe or insufficient. Reduction accuracy and fixation accuracy should be measured separately and then interpreted together.

### 5.2 TiRobot-assisted sacroiliac screw fixation

TiRobot-assisted percutaneous screw fixation is supported by a larger evidence base than RAFR. A 2022 meta-analysis reported favorable radiation, guidewire-attempt, blood-loss, screw-accuracy, and Majeed outcomes, without clear differences in operative time, healing time, or reduction accuracy (22). A 2026 meta-analysis of 11 comparative studies similarly evaluated perioperative, radiographic, functional, complication, cost, and resource outcomes, but heterogeneity and the quality of included studies still limit causal certainty (6).

These findings concern navigated fixation, not closed reduction. TiRobot and the currently reported Rossum-based system perform different tasks and should be evaluated separately before any combined-workflow inference is made.

Small clinical series in patients with sacral variations further show the value of robot-assisted trajectory control. Yang et al. (23) reported TiRobot-assisted percutaneous sacroiliac cannulated screw fixation for posterior ring injury with sacral variation, with favorable screw accuracy and no reported vascular or nerve injury in a small cohort. This scenario is clinically important because sacral variation is exactly the anatomical situation in which conventional fluoroscopic screw placement can be difficult.

### 5.3 Robot-assisted screw placement vs. manual screw placement

A 2024 study reported associations between robot-assisted screw placement and fewer fluoroscopic exposures, lower early pain, shorter hospital stay, and higher early Majeed scores than manual placement; longer-term functional differences were less clear (24).

This pattern is compatible with technical or early-recovery benefit, but it does not establish durable functional superiority. Future comparative studies should control for baseline injury, reduction method, fixation construct, rehabilitation, and surgeon experience.

### 5.4 O-arm, 3D navigation, and trans-sacral fixation

O-arm and three-dimensional navigation systems represent a related technology lineage. Their main contribution is intraoperative visualization, registration, and screw trajectory control. Coste et al. reported O-arm CT-guided navigation for percutaneous iliosacral screw fixation in unstable pelvic ring lesions (25). Other experiences with O-arm or 3D-navigated percutaneous pelvic screws support the feasibility of navigation-assisted fixation (26, 27).

Trans-sacral trans-iliac screws are particularly relevant in geriatric or osteoporotic pelvic ring injuries because they may provide longer bone purchase and bilateral posterior ring stabilization. Kramer et al. reported percutaneous 3D-navigated screw fixation for sacral fragility fractures, including extensive use of trans-sacral screws, but also noted revision surgery for screw loosening in some patients (28). Navigation improves placement; bone quality and construct stability still determine whether fixation will hold.

### 5.5 Escalation strategies for complex posterior ring instability

For highly unstable sacral fractures, spinopelvic dissociation, or cases in which iliosacral or trans-sacral fixation is inadequate, lumbopelvic fixation or S2-alar-iliac constructs may be considered as escalation strategies. They should not be framed as routine substitutes for standard minimally invasive posterior ring fixation. Biomechanical work suggests relevance for sacral fracture fixation, but clinical generalization remains limited (29).

## 6 Postoperative audit and outcome standardization

### 6.1 Rationale for standardized assessment

A patient-specific surgical pathway should end with postoperative audit. Evaluation should distinguish technical feasibility, radiographic reduction, implant accuracy, clinical effectiveness, and safety, while also reporting invasiveness, radiation, recovery, implementation burden, and long-term patient-centered outcomes.

This need is especially important in robotic pelvic fracture surgery. If reduction quality, screw accuracy, residual displacement, function, and complications are defined differently, results cannot be compared across centers. Although no consensus core outcome set currently exists for RAFR studies, a shared minimum dataset would make future evidence more interpretable. Table 2 presents an author-proposed candidate core dataset; it is not a consensus core outcome set.

**Table 2:** Author–proposed candidate core reporting domains for future studies of robot–assisted pelvic fracture reduction.

Domain	Essential variables	Method/time point	Purpose
Baseline phenotype and displacement	Tile/AO and Young–Burgess classification; posterior/anterior injury; frailty; bone quality; maximum translation, vertical migration, and rotation	Preoperative CT and radiographs using prespecified landmarks	Allows risk adjustment and comparison of reduction difficulty
Reduction quality	Residual CT translation and rotation; Matta E/G/F/P reported separately	Immediate postoperative CT; state planes, landmarks, thresholds, assessor, and imaging source	Separates quantitative 3D alignment from historical grading
Implant accuracy	Cortical/foraminal/intra–articular breach; guidewire adjustment; screw revision	Immediate postoperative CT using a prespecified grading rule	Audits fixation independently from reduction
Robotic execution and workflow	Planned–vs.–achieved motion; force/torque; attempts; setup, registration, reduction, fixation, and total OR time	Time–stamped system and operating–room logs; case sequence	Defines control accuracy, failure modes, and learning curve
Radiation and invasiveness	Dose–area product; cumulative air kerma; fluoroscopy time/events; staff dose; incision, blood loss, transfusion	Automatic imaging–system export, dosimeter where feasible, and operative record	Avoids using fluoroscopy count alone as a radiation surrogate
Recovery and function	Pain, mobilization, hospital stay, Majeed score, walking, work/independent living, quality of life	Discharge; 6 weeks; 3, 6, 12, and ≥24 months with denominators	Captures early recovery and durable patient–centered outcomes
Safety and reoperation	Neurovascular injury; infection; loss of reduction; implant failure; revision; device–related events; conversion	Prospective definitions and active surveillance at 30/90 days and final follow–up	Establishes clinical and device safety boundaries
Completeness and reliability	Eligible/enrolled/analyzed numbers; attrition; missing data; inter– and intra–assessor agreement	Flow accounting; independent assessors; ICC or kappa as appropriate	Prevents selective outcome reporting and quantifies measurement error

AO, Arbeitsgemeinschaft für Osteosynthesefragen; CT, computed tomography; E/G/F/P, excellent/good/fair/poor; ICC, intraclass correlation coefficient; OR, operating room. These domains are proposed by the authors to support comparability; they are not an established consensus core outcome set.

### 6.2 Radiographic reduction

Matta criteria remain widely used for pelvic ring reduction grading (30), but authors should state the imaging modality and anatomical measurement used. Terms should be standardized as excellent, good, fair, or poor; the combined category should be reported as ‘excellent or good,' not as a substitute for the individual grades. Because RAFR targets three-dimensional deformity, quantitative postoperative CT should accompany radiographic grading whenever feasible.

Fluoroscopic inlet and outlet views remain useful intraoperatively, but CT offers a more objective assessment of posterior ring alignment, sacroiliac congruity, screw position, cortical breach, and residual displacement. Future RAFR studies should report both Matta grading and quantitative CT-based displacement whenever possible.

### 6.3 Residual displacement and functional outcome

The relationship between residual displacement and function remains unsettled. Some studies suggest that residual displacement up to 10 mm may still be compatible with acceptable functional outcomes, whereas other analyses associate smaller residual displacement, particularly below 5 mm, with better results (31, 32).

A practical reporting strategy is to present displacement as a continuous measurement in millimeters and also report thresholds such as < 5 mm, 5–10 mm, and >10 mm. In RAFR-assisted surgery, < 5 mm may be an aspirational benchmark, while < 10 mm may remain acceptable in selected patients depending on injury pattern, physiological status, and recovery trajectory.

### 6.4 Implant accuracy and safety

Postoperative audit should include screw position. For iliosacral, trans-sacral, anterior column, pubic ramus, LC-2, or other percutaneous screws, authors should report cortical breach, foraminal violation, intra-articular placement, guidewire adjustment, screw revision, and neurovascular complications.

In combined reduction-robot and TiRobot workflows, reduction and implant accuracy must be reported separately. Reduction should use prespecified CT landmarks and residual-displacement methods; fixation should report screw position, cortical or foraminal breach, guidewire adjustments, and revision. Success in one domain must not obscure failure in another.

### 6.5 Functional and patient-centered outcomes

The Majeed pelvic score remains one of the most widely used functional measures in pelvic ring injury studies (33). It should not be the only outcome, particularly in older patients, polytrauma patients, or those with neurological injury.

Future studies should supplement the Majeed score with VAS pain, walking ability, time to mobilization, return to work or independent living, EQ-5D, SF-36, and geriatric mobility outcomes when appropriate. In osteoporotic pelvic fractures, clinically meaningful endpoints may include pain relief, transfer ability, walking independence, discharge destination, and avoidance of immobility-related complications.

## 7 Potentially suitable scenarios and relative limitations

The scenarios in Supplementary Table S2 are separated by evidence maturity. Direct RAFR evidence is limited to model validation, early unstable Tile B/C cohorts, one non-randomized comparison, and a single-center FFP type III series (7–13, 20). Damage-control exclusions, equivocal instability, mirror-template limitations, and some fixation considerations derive from broader pelvic trauma, EUA, digital-planning, and navigation literature (4, 16–19, 28, 34–37). The table is an evidence-maturity map, not a set of clinical indications or recommendations.

Robot-assisted closed reduction should not be presented as a universal solution or as an emergency substitute for established damage-control principles. In hemodynamically unstable pelvic injuries, early care must prioritize hemorrhage control, pelvic binding, external fixation, C-clamp application when appropriate, preperitoneal packing, and/or angioembolization according to local protocols, consistent with pelvic trauma guidelines and standard staged management principles (36). RAFR is better positioned as a definitive or staged reconstructive option after physiological stabilization.

Practical selection also depends on whether the workflow can be executed safely. Severe bilateral posterior comminution, pre-existing deformity, or previous pelvic surgery may invalidate mirror-based planning (16–18). Open pelvic fractures, extensive degloving, and bone unable to support holding pins are relative limitations extrapolated from damage-control and workflow safety principles rather than direct RAFR evidence (36).

Force-controlled stress testing for equivocal instability is a research hypothesis. EUA literature shows the clinical problem but does not validate a robotic diagnostic threshold (34, 35). Any study should predefine force vector, magnitude, duration, displacement threshold, imaging method, repeatability, stopping rules, and reference standard. Until such validation exists, robotic stress testing is not an established indication.

A 2024 case series provides emerging direct evidence for 15 displaced FFP type III injuries treated with robot-assisted reduction and fixation, with 6-month follow-up (20). This extends feasibility beyond high-energy Tile B/C injuries, but the uncontrolled single-center design does not establish superiority or rare-event safety. Bone quality, pin purchase, fixation construct, augmentation strategy, and mobilization remain decisive (20, 28, 37).

## 8 Challenges and future research agenda

### 8.1 Evidence gap

The principal limitation is evidence quality. Most RAFR reports are single-center, retrospective, early-phase, or case-series studies. A non-randomized comparison cannot remove confounding by injury pattern, displacement, time to surgery, surgeon experience, fixation strategy, rehabilitation, or learning curve. Possible overlap among reports 10–12 further reduces the apparent independent sample size.

Evidence for screw-placement robotics is more mature and includes multiple meta-analyses (6, 22), but it cannot be used as surrogate proof for reduction robotics. Current RAFR data support selected technical feasibility and early radiographic outcomes only.

The next step is to move beyond feasibility. Independent multicenter prospective studies should compare RAFR-assisted closed reduction plus navigated fixation with conventional closed or limited-open reduction plus percutaneous fixation. Stratification by Tile/AO type, Young-Burgess mechanism, posterior ring injury, sacral morphology, age, bone quality, injury-to-surgery interval, and baseline displacement will be necessary.

### 8.2 Technical gap

Technical generalizability is another challenge. Mirror-template planning assumes that the contralateral hemipelvis is intact and representative. This assumption may fail in bilateral posterior ring injuries, severe comminution, pre-existing deformity, previous pelvic surgery, or combined pelvic-acetabular injuries.

Registration accuracy and force control are equally important. Intraoperative CBCT registration, optical tracking, tracker fixation, holding-pin stability, elastic traction, and robotic arm execution are all potential sources of error. Severe osteoporosis may impair pin purchase, and soft-tissue resistance may make a planned path unsafe or incomplete.

Future RAFR studies should report registration error; planned-vs.-achieved translation and rotation; maximum and time-integrated force/torque; threshold exceedance; attempts; manual unlocking or assistance; predefined stop and abort criteria; conversion; and device-related events. Diagnostic stress-testing studies additionally require test-retest reliability and agreement with an independent clinical reference standard.

### 8.3 Implementation gap

Implementation is a third challenge. RAFR requires preoperative planning, intraoperative imaging, registration, tracker placement, robotic setup, team coordination, sterilization, and a backup conversion plan. These steps may increase operative complexity during early adoption.

Workflow and learning curve should be measured by planning, setup, registration, reduction, fixation, and total operating-room time; staffing; troubleshooting; conversion; and device-related delays. Case-sequence plots and cumulative-sum analysis should be considered so that early setup burden is not conflated with mature performance.

Cost-effectiveness remains unresolved. Robotic systems require capital investment, maintenance, technical support, and team training. A robotic pathway may be justified if it reduces complications, radiation exposure, blood loss, hospital stay, revision surgery, or long-term disability, but these assumptions require prospective health-economic evaluation.

### 8.4 Standardization gap

Standardization is the fourth challenge. Current studies vary in their reporting of reduction quality, screw accuracy, radiation exposure, operative time, functional outcome, complications, and follow-up duration. This heterogeneity limits comparison and evidence synthesis.

Until a core outcome set is developed, studies should report baseline displacement; a reproducible CT residual-displacement method; Matta grade with imaging source; independent-assessor reliability; implant breach; radiation dose-area product and cumulative air kerma; staff dose where available; blood loss; incision length; operative time; robotic force/torque and abort events; complications; reoperation; healing; fixed-time-point Majeed, pain, mobility, and quality-of-life outcomes; attrition; and learning-curve sequence. An extended author-proposed reporting set is provided in Supplementary Table S3.

Indication reporting should also be standardized. Authors should specify fracture classification, displacement severity, reducibility, sacral dysmorphism, bone quality, contralateral template availability, soft-tissue condition, and reasons for excluding robotic reduction. Without these details, it will remain difficult to identify which patients benefit most.

### 8.5 Future research agenda

The next phase should move from device-centered reports to independently governed, patient- and outcome-centered studies. The priority is not whether a robot can complete a planned motion, but whether appropriately selected patients experience better validated outcomes without unacceptable harm or implementation burden.

Priority questions include patient selection, failure criteria for conventional closed reduction, target CT displacement, conversion to limited-open or open reduction, long-term function and quality of life, device-related harm, learning curve, safe force thresholds, cost-effectiveness, and external reproducibility. Prospective multicenter registries should precede or support adequately powered comparative trials. Force-controlled stress testing should remain a separate diagnostic research program until thresholds are validated.

Future technology may incorporate automated segmentation, AI-assisted reduction planning, intraoperative safety monitoring, augmented or mixed reality visualization, digital twin modeling, and remote robotic workflows. These tools should be framed as surgeon-supervised decision support rather than substitutes for surgical judgment. The long-term goal is an intelligent pathway that quantifies deformity, proposes safe reduction trajectories, monitors force and position, assists fixation, and generates standardized postoperative audit data.

## 9 Conclusion

Pelvic fracture robotics is expanding from navigated implant placement to robot-assisted closed reduction. The clinically relevant endpoint is not robot use itself, but a reproducible sequence of reduction, fixation, and audit.

The currently reported Rossum-based workflow links digital planning to controlled reduction and navigated fixation. Present evidence supports technical feasibility and early radiographic acceptability in selected unstable and fragility pelvic ring injuries; it does not establish comparative effectiveness, long-term safety, durable function, or cost-effectiveness.

Adoption should remain jurisdiction-appropriate, indication-specific, and prospectively audited. Independent multicenter studies with transparent cohort accounting, standardized CT and functional endpoints, assessor reliability, complete safety reporting, and health-economic evaluation are required before routine guideline-level use can be justified.

## References

[1] Alton TB Gee AO. Young and Burgess classification of pelvic ring injuries. Clin Orthop Relat Res. (2014) 472:2338–42. doi: 10.1007/s11999-014-3693-8 24867452 PMC4079881

[2] Wu B Wang G Zheng J. Robot-assisted fracture fixation for pelvic fractures: a scoping review of emerging technologies. Front Surg. (2025) 12:1559419. doi: 10.3389/fsurg.2025.1559419 41164385 PMC12558912

[3] Kou W Zhou P Lin J Kuang S Sun L. Technologies evolution in robot-assisted fracture reduction systems: a comprehensive review. Front Robot AI. (2023) 10:1315250. doi: 10.3389/frobt.2023.1315250 38077454 PMC10702561

[4] Giannoudis PV Tzioupis CC Pape HC Roberts CS. Percutaneous fixation of the pelvic ring: an update. J Bone Joint Surg Br. (2007) 89:145–54. doi: 10.1302/0301-620X.89B2.18551 17322425

[5] Le Baron M Pithioux M Candoni S Flecher X. How can technology improve acetabular and pelvic fractures management in the operating room? A systematic review. Orthop Surg. (2026) 18:915–31. doi: 10.1111/os.70297 41960722 PMC13139054

[6] Yin Y Shen G Wang R Chen H Wang K Chen Y . Comprehensive clinical outcomes of TiRobot-assisted minimally invasive surgery for pelvic fractures: a meta-analysis. J Robot Surg. (2026) 20:213. doi: 10.1007/s11701-026-03212-z 41629723

[7] Zhao C Wang Y Wu X Zhu G Shi S. Design and evaluation of an intelligent reduction robot system for the minimally invasive reduction in pelvic fractures. J Orthop Surg Res. (2022) 17:205. doi: 10.1186/s13018-022-03089-2 35379278 PMC8981738

[8] Ge Y Zhao C Wang Y Wu X. Robot-assisted autonomous reduction of a displaced pelvic fracture: a case report and brief literature review. J Clin Med. (2022) 11:1598. doi: 10.3390/jcm11061598 35329924 PMC8950953

[9] Zhao C Cao Q Sun X Wu X Zhu G Wang Y. Intelligent robot-assisted minimally invasive reduction system for reduction of unstable pelvic fractures. Injury. (2023) 54:604–14. doi: 10.1016/j.injury.2022.11.001 36371315

[10] Wu Z Dai Y Zeng Y. Intelligent robot-assisted fracture reduction system for the treatment of unstable pelvic fractures. J Orthop Surg Res. (2024) 19:271. doi: 10.1186/s13018-024-04761-5 38689343 PMC11059586

[11] Dai Y Zeng Y Wu Z Zhao C Wang J Wu X. Clinical efficacy of intelligent pelvic fracture reduction robot combined with TiRobot in treating unstable pelvic fractures. J Cap Med Univ. (2024) 45:763–72. doi: 10.21203/rs.3.rs-4159758/v1

[12] Dai Y Zeng Y Shi H Zhou J Zhao C. Efficacy analysis of robot-assisted minimally invasive surgery for the treatment of unstable pelvic fractures. BMC Surg. (2025) 25:296. doi: 10.1186/s12893-025-03065-7 40670930 PMC12269219

[13] Fan X He J Qi B Zhang H Li G Liu X . Intelligent robot-assisted fracture reduction for pelvic fractures: a clinical study. Front Med. (2026) 13:1744048. doi: 10.3389/fmed.2026.1744048 41788710 PMC12958122

[14] Shen L Xue X Ping Y Song Z Zhong C Su G . Evolution of the reduction technique for unstable pelvic ring fractures: a narrative review. Eur J Med Res. (2025) 30:335. doi: 10.1186/s40001-025-02570-y 40287764 PMC12032693

[15] Ge L Wang L Duan L Zhang T. Rebuilding the pelvis: advances in robotic-assisted management of complex pelvic fractures. Comput Assist Surg. (2026) 31:2615212. doi: 10.1080/24699322.2026.2615212 41543252

[16] Ead MS Westover L Polege S McClelland S Jaremko JL Duke KK. Virtual reconstruction of unilateral pelvic fractures by using pelvic symmetry. Int J Comput Assist Radiol Surg. (2020) 15:1267–77. doi: 10.1007/s11548-020-02140-z 32249403

[17] Zhao C Guan M Shi C Zhu G Gao X Wang Y . Automatic reduction planning of pelvic fracture based on symmetry. Comput Methods Biomech Biomed Eng Imaging Vis. (2022) 10:577–84. doi: 10.1080/21681163.2021.2012830

[18] Liu Y Yibulayimu S Sang Y Zhu G Shi C Liang C . Preoperative fracture reduction planning for image-guided pelvic trauma surgery: a comprehensive pipeline with learning. Med Image Anal. (2025) 102:103506. doi: 10.1016/j.media.2025.103506 39999763

[19] Liu J Ge Y Yibulayimu S Liu Y Wu X Wang Y . Patient-specific musculoskeletal modeling to enhance preoperative planning for pelvic fracture reduction. Commun Med. (2025) 5:532. doi: 10.1038/s43856-025-01238-2 41258529 PMC12738769

[20] Zhao C Xiao H Cao Q Ge Y Li Y Wang Y . Innovative development of robot reduction system in geriatric pelvic fractures: a single-center case series in Beijing, China. J Orthop Transl. (2024) 49:283–8. doi: 10.1016/j.jot.2024.08.023 PMC11555238 39534853

[21] National Medical Products Administration. Robot-Assisted Fracture Reduction System for Pelvic Fracture Approved for Marketing. Published December 11, 2023. Available online at: https://english.nmpa.gov.cn/2023-12/11/c_964004.htm (accessed May 31, 2026).

[22] Zhao C Zhu G Wang Y Wu X. TiRobot-assisted versus conventional fluoroscopy-assisted percutaneous sacroiliac screw fixation for pelvic ring injuries: a meta-analysis. J Orthop Surg Res. (2022) 17:525. doi: 10.1186/s13018-022-03420-x 36471345 PMC9721051

[23] Yang C Liu G Tang J Li G Qin X Hu J. TiRobot-assisted percutaneous sacroiliac cannulated screw fixation for posterior pelvic ring injury with sacral variations. Zhongguo Xiu Fu Chong Jian Wai Ke Za Zhi. (2022) 36:940–5. doi: 10.7507/1002-1892.202204043 35979783 PMC9379457

[24] Wang M Zheng S Zhang Y Lu J. Analysis of the therapeutic efficacy of robot-assisted percutaneous screw fixation in the minimally invasive treatment of pelvic fractures. Front Surg. (2024) 11:1392719. doi: 10.3389/fsurg.2024.1392719 39022596 PMC11251939

[25] Coste C Asloum Y Marcheix PS Dijoux P Charissoux JL Mabit C. Percutaneous iliosacral screw fixation in unstable pelvic ring lesions: the interest of O-arm CT-guided navigation. Orthop Traumatol Surg Res. (2013) 99:S273–8. doi: 10.1016/j.otsr.2013.03.002 23639760

[26] Ciolli G Caviglia D Vitiello C Cappuccio M Ghermandi R Gasbarrini A . Navigated percutaneous screw fixation of the pelvis with O-arm 2: two years' experience. Med Glas. (2021) 18:309–15. doi: 10.17392/1326-21 33480224

[27] Florio M Capasso L Olivi A Vitiello C Leone A Liuzza F. 3D-navigated percutaneous screw fixation of pelvic ring injuries: a pilot study. Injury. (2020) 51:S28–33. doi: 10.1016/j.injury.2020.07.025 32723529

[28] Kramer A Naisan M Kindel S Richter M Ringel F Hartung P. Retrospective evaluation of percutaneous 3D-navigated screw fixation for fragility fractures of the sacrum: technical notes and four-year experience. Sci Rep. (2023) 13:12254. doi: 10.1038/s41598-023-39165-8 37507446 PMC10382507

[29] Zheng J Feng X Xiang J Liu F Leung FKL Chen B. S2-alar-iliac screw and S1 pedicle screw fixation for the treatment of non-osteoporotic sacral fractures: a finite element study. J Orthop Surg Res. (2021) 16:727. doi: 10.1186/s13018-021-02805-8 34717718 PMC8557573

[30] Matta JM Saucedo T. Internal fixation of pelvic ring fractures. Clin Orthop Relat Res. (1989) 242:83–97. doi: 10.1097/00003086-198905000-00009 2706863

[31] Gänsslen A Lindahl J Krappinger D Lindtner RA Staresinic M. Outcome of pelvic ring injuries. Arch Orthop Trauma Surg. (2025) 145:47. doi: 10.1007/s00402-024-05610-0 PMC11649792 39680172

[32] Kataria M Aggarwal S Bachhal V Jindal K Appajigowda A. Does the residual displacement of pelvic ring affect the functional outcome in pelvic ring injuries? Int J Burns Trauma. (2023) 13:44–50. 37215508 PMC10195215

[33] Majeed SA. Grading the outcome of pelvic fractures. J Bone Joint Surg Br. (1989) 71:304–6. doi: 10.1302/0301-620X.71B2.2925751 2925751

[34] Sagi HC Coniglione FM Stanford JH. Examination under anesthetic for occult pelvic ring instability. J Orthop Trauma. (2011) 25:529–36. doi: 10.1097/BOT.0b013e31822b02ae 21857421

[35] Keltz E Keren Y Jain A Stephens T Rovitsky A Ghrayeb N . Surgical stabilisation in equivocal pelvic ring injuries - Into the grey zone. Injury. (2023) 54:110887. doi: 10.1016/j.injury.2023.110887 37453290

[36] Coccolini F Stahel PF Montori G Biffl W Horer TM Catena F . Pelvic trauma: WSES classification and guidelines. World J Emerg Surg. (2017) 12:5. doi: 10.1186/s13017-017-0117-6 28115984 PMC5241998

[37] Rommens PM Hofmann A. Comprehensive classification of fragility fractures of the pelvic ring: recommendations for surgical treatment. Injury. (2013) 44:1733–44. doi: 10.1016/j.injury.2013.06.023 23871193

