# Supplementary material for: Robot-assisted closed reduction and navigated fixation for pelvic ring injuries: a focused narrative review of a reduction-fixation-audit framework
Source: Front Med (Lausanne). 2026 Aug 27;13:1883771. doi: 10.3389/fmed.2026.1883771 (PMC13560486; doi:10.3389/fmed.2026.1883771)
Supplement: Supplementary file 1 [file Table_1.docx]

# Supplementary material

**Supplementary Table S1. Neutral comparison of the scope and limitations of recent reviews.**

| **Study/design** | **Primary scope** | **Relevant strength** | **Gap relative to the present review question** |
| --- | --- | --- | --- |
| Kou et al., 2023 (2); comprehensive review | Engineering evolution of robot-assisted fracture-reduction systems | Broad technical foundation | Not pelvic-ring-specific; limited clinical outcome audit |
| Shen et al., 2025 (32); narrative review with systematic search | Evolution of unstable pelvic ring reduction techniques | Directly addresses reduction evolution | Search ended April 2024; limited emphasis on cohort independence and standardized outcomes |
| Wu et al., 2025 (1); scoping review | Robot-assisted pelvic fracture fixation | Summarizes fixation technologies | Reduction is mainly indirect; reduction and implant outcomes are not consistently separated |
| Le Baron et al., 2026 (35); systematic review | Operating-room technologies for pelvic and acetabular fractures | Broad technology mapping | Mixed pelvic/acetabular scope; not specific to RAFR |
| Ge et al., 2026 (34); narrative review | Robotic management of complex pelvic fractures | Contemporary robotic overview | Heterogeneous technologies; limited emphasis on evidence maturity and possible cohort overlap |

Note: RAFR, robot-assisted fracture reduction. The comparison identifies differences in scope and does not imply methodological superiority of the present review.

**Supplementary Table S2. Evidence-maturity map of reported and hypothetical clinical scenarios.**

| **Clinical scenario** | **Evidence source/type** | **Permissible inference** | **Limitations** |
| --- | --- | --- | --- |
| Fresh unstable Tile B injury | Direct early case-series evidence (10-14) | Technical feasibility may be studied in selected fresh, closed, reducible injuries after physiological stabilization. | No guideline-level indication; incremental benefit over standard closed reduction unknown. |
| Fresh unstable Tile C injury | Direct early case-series evidence (10-14) | Selected multidirectional reductions may be technically feasible with adequate traction, pin control, and a backup plan. | Severe displacement, comminution, or delay may require limited-open or open assistance. |
| Displaced geriatric FFP | One prospective single-center series of FFP type III injuries (31) | Preliminary feasibility only in a selected cohort with adequate pin purchase and fixation planning. | Uncontrolled 15-patient evidence; comparative and long-term benefit unknown. |
| Equivocal instability | Indirect EUA literature and a diagnostic hypothesis (27,28) | Force-controlled stress imaging is hypothesis-generating research only. | No validated robotic diagnostic threshold; no clinical recommendation can be made. |
| Polytrauma after resuscitation | Indirect pelvic-trauma guidance plus RAFR series (10-14,29) | A definitive-stage research pathway may be considered only after physiological stabilization. | Not appropriate for hemodynamically unstable hemorrhagic injury. |
| Sacral dysmorphism or narrow corridors | Direct/indirect evidence for navigated fixation (3,15,16,21,36) | Navigation may assist post-reduction screw-trajectory planning. | Fixation-navigation evidence does not establish a reduction benefit. |
| Bilateral posterior injury | Planning studies and indirect evidence (5-7,33) | Alternative validated target models are required before study. | Contralateral mirror-template assumptions may be invalid. |
| Open fracture or degloving | Damage-control guidance; no direct RAFR evidence (29) | No efficacy inference is permissible in acute contaminated management. | Pins, registration, and workflow may be unsafe; soft-tissue and hemorrhage priorities dominate. |
| Chronic malunion or irreducibility | No direct RAFR effectiveness evidence | At most a research adjunct to individualized osteotomy/open-reduction planning. | Closed robotic motion may be inadequate or harmful. |
| Pre-existing deformity or prior pelvic surgery | Planning studies and indirect evidence (5-7,33) | A validated non-mirror patient-specific target would be required. | Pelvic asymmetry may invalidate mirror reconstruction. |
| Spinopelvic dissociation | Biomechanical/indirect evidence (22) | No clinical RAFR effectiveness inference; fixation strategy remains primary. | Lumbopelvic or triangular fixation may be required. |

Note: CT, computed tomography; EUA, examination under anesthesia; FFP, fragility fracture of the pelvis; RAFR, robot-assisted fracture reduction. Categories describe evidence maturity, not formal indications. Hypothetical scenarios are research questions and should not be interpreted as clinical recommendations.

**Supplementary Table S3. Extended author-proposed candidate reporting domains for future RAFR studies.**

| **Domain** | **Priority** | **Candidate variables** | **Suggested method/time point** |
| --- | --- | --- | --- |
| Baseline injury severity | Essential | Tile/AO; Young-Burgess; posterior/anterior injury; physiology, frailty, and bone quality | Preoperative CT, radiographs, and clinical record; preoperative |
| Baseline displacement | Essential | Maximum translation, vertical migration, rotation, and 3D point-cloud deviation | Prespecified CT landmarks and planes; preoperative |
| Reduction quality | Essential | Residual CT translation/rotation; mean, maximum, distribution, and thresholds | Prespecified CT landmarks; immediate postoperative |
| Reduction grade | Essential | Matta excellent/good/fair/poor reported separately | State CT versus radiograph and exact rule; immediate postoperative |
| Implant accuracy | Essential | Cortical breach, foraminal violation, intra-articular placement, screw-position grade | Postoperative CT; immediate postoperative |
| Surgical invasiveness | Extended | Incision length, blood loss, transfusion, and soft-tissue complications | Operative record and early follow-up |
| Radiation exposure | Essential | Dose-area product, cumulative air kerma, fluoroscopy time/events, and staff dose | Automatic system export and dosimeter where feasible; intraoperative |
| Workflow/learning curve | Essential | Planning, setup, registration, reduction, fixation, total OR time, staffing, and case sequence | Time-stamped record; CUSUM/prespecified sequence analysis |
| Early recovery | Extended | VAS pain, mobilization, length of stay, and discharge destination | Clinical record; discharge and 2-6 weeks |
| Function | Essential | Majeed score, walking, work, and independent living with denominator/attrition | 3, 6, 12, and >=24 months |
| Quality of life | Extended | EQ-5D, SF-36, or another validated patient-reported outcome | 6 and 12 months |
| Safety | Essential | Neurovascular injury, infection, loss of reduction, implant failure, revision, and device events | Prospective definitions; intraoperative, 30/90 days, and final follow-up |
| Implementation | Essential | Manual assistance/unlocking, stop/abort, conversion, malfunction, and troubleshooting | Time-stamped operating-room record; intraoperative |
| Measurement reliability | Essential | Inter- and intra-assessor agreement | Two independent assessors; ICC for continuous and kappa for categorical outcomes |
| Robotic execution | Essential | Planned-versus-achieved motion; maximum force/torque; threshold exceedance | System log with prespecified thresholds and override documentation |
| Economic evaluation | Exploratory | Capital, maintenance, staff, consumables, OR time, hospital use, revision, and quality-adjusted life-years | Prospective resource capture; prespecified health-economic horizon |

Note: AO, Arbeitsgemeinschaft für Osteosynthesefragen; CT, computed tomography; CUSUM, cumulative sum; EQ-5D, EuroQol five-dimension instrument; ICC, intraclass correlation coefficient; OR, operating room; RAFR, robot-assisted fracture reduction; SF-36, 36-item Short Form Health Survey; VAS, visual analogue scale. Priority assignments are author-proposed and require external consensus and validation.
